# Hidden hunger in South Asia: a review of recent trends and persistent challenges

**DOI:** 10.1017/S1368980017003202

**Published:** 2017-12-19

**Authors:** Kassandra L Harding, Víctor M Aguayo, Patrick Webb

**Affiliations:** 1 Friedman School of Nutrition Science and Policy, Tufts University, 150 Harrison Avenue, Boston, MA 02111, USA; 2 UNICEF Nutrition Programme, Programme Division, New York, NY, USA

**Keywords:** Micronutrients, Hidden Hunger, Undernutrition, Policy, South Asia

## Abstract

‘Hidden hunger’ is a term used to describe human deficiencies of key vitamins and minerals, also known as micronutrients. While global in scale, the prevalence of micronutrient deficiencies is particularly high in South Asia despite recent successes in economic growth, agricultural output and health care. The present paper reviews the most recent evidence on patterns and trends of hidden hunger across the region, with a focus on the most significant deficiencies – iodine, Fe, vitamin A and Zn – and interprets these in terms of health and economic consequences. The challenge for South Asian policy makers is to invest in actions that can cost-effectively resolve chronic nutrient gaps facing millions of households. Appropriate solutions are available today, so governments should build on evidence-based successes that combine targeted health system delivery of quality services with carefully designed multisector actions that help promote healthier diets, reduce poverty and ensure social protection simultaneously.

‘Hidden hunger’ is a term used to describe human deficiencies in essential vitamins and minerals, also known as micronutrients. Micronutrient deficiencies affect an estimated two billion people, or almost one-third of the world’s population^(^ ^1^ ^)^. Iodine, Fe, vitamin A and Zn deficiencies are the four micronutrient deficiencies of greatest public health concern globally, due to their high prevalence and associated health and developmental consequences. Roughly one-third of children aged 6–59 months (children <5 years) in low- and middle-income countries suffer from vitamin A deficiency (VAD)^(^ ^2^ ^)^ and 18 % of children <5 years have Fe-deficiency anaemia^(^ ^3^ ^)^. Similarly, 30 % of people worldwide suffer from insufficient iodine intake^(^ ^4^ ^)^ and 17 % from inadequate Zn intake^(^ ^5^ ^)^. While there is overlap across such deficiencies at both population and individual levels, and the full extent of multiple deficiencies remains poorly documented, hidden hunger remains a global challenge.

That said, micronutrient deficiencies are more severe and affect more people in poorer regions of the world. In 2013, approximately 1·7 % (95 % credible interval 1·0, 2·6 %) of all deaths among children <5 years in low- and middle-income countries were attributable to VAD, with 95 % of these deaths occurring in South Asia and sub-Saharan Africa^(^ ^2^ ^)^. Although the prevalence of insufficient iodine intake in South Asia is comparable to the global average (32 % compared with 30 %)^(^ ^4^ ^)^, anaemia among pregnant women (52 %) and children <5 years (58 %) exceeds the global prevalence (38 and 43 %, respectively), as does inadequate Zn intake (at 30 % *v*. the global average of 17 %)^(^ ^3^ ^,^ ^6^ ^)^.

This situation is of great concern for South Asia’s future since it is associated with significant health consequences that translate into huge economic losses. Despite recent progress across much of South Asia in terms of economic growth, agricultural output and exports, poverty reduction and even some indicators of improved child nutrition, the region’s burden of micronutrient deficiencies has improved little for several decades.

The present paper highlights recent empirical evidence on South Asia’s micronutrient deficiencies, documenting trends and regional patterns while pointing to various success stories around effective policies and programming. Nationally representative data from country-level reports and data sets, as well as data from peer-reviewed literature, are reviewed, compiled and presented to provide a comprehensive understanding of what is known today about hidden hunger in South Asia and where knowledge gaps persist. The most recent data available cover a period that ranges from 2005 to 2014, and are constrained by a lack of standardization of metrics and data gaps impairing consistent cross-country comparability. However, important conclusions about the state of deficiencies and the state of knowledge are drawn out, and these suggest a need for greater policy prioritization of such nutrition challenges across the whole of South Asia in coming years.

## South Asia’s nutrition challenges

South Asia presents a paradox: on the one hand, this region has become the ‘fastest growing developing region in the world’^(^ ^7^ ^)^. Economic growth remains strongest in India, and many of its neighbouring countries have recorded significant poverty reduction (Bangladesh), falling rates of child undernutrition (Nepal), declining illiteracy (Sri Lanka) and growing agricultural productivity (such as in the livestock sector in Pakistan)^(^ ^8^ ^,^ ^9^ ^)^. On the other hand, this same region is home to the world’s largest burden of malnutrition; that is, the countries of South Asia are home to the largest number of stunted children <5 years, face rapidly rising rates of overweight and obesity with a related surge in diabetes and chronic heart disease, and continue to suffer a wide range of micronutrient deficiencies^(^ ^10^ ^)^.

The four micronutrient deficiencies that we focus upon in the current review, including Fe, iodine, vitamin A and Zn, each has global significance in South Asia. Indeed, due to population size, the highest global levels of disability-adjusted life years attributable to hidden hunger are in India, Bangladesh and Pakistan^(^ ^11^ ^)^. For India alone, the burden of the top four micronutrient deficiencies has been estimated as representing an overall economic loss of 0·8 to 2·5 % of India’s Gross Domestic Product – which in 2004 was already equivalent to roughly $US 17 billion per year in monetary terms^(^ ^12^ ^)^.

The following sections present the latest available data on patterns and trends for hidden hunger, followed by a discussion of the policy implications associated with such significant nutrition concerns. While micronutrient deficiencies often overlap with other forms of undernutrition as well as with obesity, the nature and extent of such co-manifestations are poorly understood^(^ ^13^ ^,^ ^14^ ^)^. As a result, we limit the review to what is currently known about the prevalence and distribution of micronutrient deficiencies across South Asia. We do not seek to determine deficiencies among obese or otherwise undernourished children as co-morbidities.

## Patterns and trends of South Asia’s hidden hunger

### Zinc

**Fig. 1:**
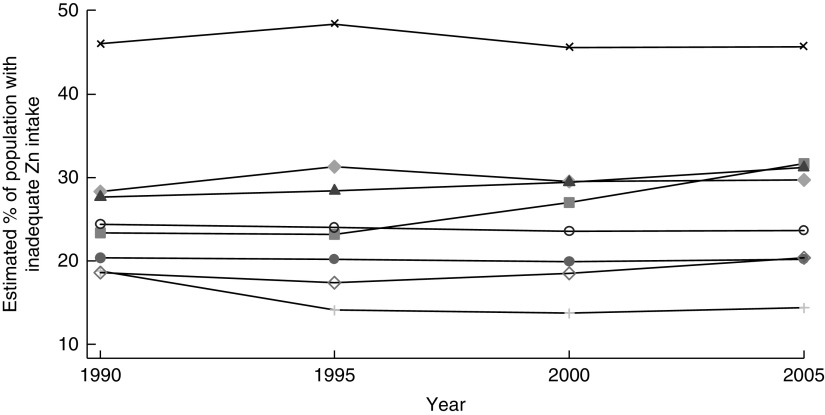
Trend in inadequate zinc intake between 1990 and 2005 in South Asia, by country: 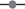, Afghanistan; 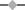, Bangladesh; 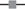, Bhutan; 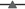, India; 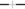, Maldives; 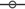, Nepal; 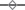, Pakistan; 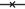, Sri Lanka. (Data from Wessells and Brown^(^ ^6^ ^)^)

Wessells *et al*. estimated that the prevalence of inadequate Zn intake in South Asia changed little between 1990 and 2005, although it increased in Bangladesh, Bhutan, India and Pakistan (Fig. 1)^(^ ^5^ ^)^. While these estimates are limited by the modelling approach adopted of using FAO food balance sheet data and must be clearly understood as estimates of inadequate intake *v*. Zn status, they provide estimates across the region that can be compared with the rest of the world and tracked across time. Using similar data, a recent analysis of the impact of rising concentrations of atmospheric CO_2_ on Zn deficiency has suggested that climate change could put an additional 138 million people at risk for Zn deficiency by 2050: 48 million of the affected people would live in India under a business-as-usual scenario^(^ ^15^ ^)^.

Other measures of Zn deficiency based on Zn status have also been used to document South Asia’s burden and, while not strictly comparable, in general these also report very high rates in this region compared with other low-income regions. For example, serum Zn concentration, which provides a more ‘direct’ measure of Zn deficiency, has been reported at the national level in Afghanistan, Bangladesh, Pakistan and Sri Lanka among women and children. Measured this way, Zn deficiency defined as serum Zn concentration <60 µg/dl is reported to be prevalent among 15 % of children <5 years in Afghanistan^(^ ^16^ ^)^. In Bangladesh and Pakistan, the prevalence is higher at 45 and 39 %, respectively. By contrast, in Sri Lanka, where Zn deficiency was defined as <65 µg/dl in the morning and <57 µg/dl in the afternoon, the prevalence was found to be substantially lower at 5·1 %^(^ ^17^ ^)^.

Such comparisons should be treated cautiously given methodological differences (e.g. the Bangladesh survey adjusted for inflammatory markers and Sri Lanka excluded children with high C-reactive protein levels^(^ ^18^ ^,^ ^19^ ^)^). In Bangladesh, deficiencies are higher in slums (52 %) and rural areas (49 %) compared with urban areas (30 %)^(^ ^19^ ^)^. Conversely, the prevalence did not differ between urban and rural areas in Pakistan, although it ranged from 33 to 47 % by province and changed little over the last decade^(^ ^18^ ^)^. Similarly, stunting among children <5 years is sometimes used as a non-clinical indicator of Zn deficiency and is widely collected in national health surveys such as the Demographic and Health Surveys and many national nutrition surveys. Given the widespread and relatively frequent assessment of stunting in populations, these data are typically used as an indicator of Zn deficiency, such as the case with the ‘hidden hunger indices’ generated by Muthayya *et al*.^(^ ^11^ ^)^. Using the metric of stunting, Pakistan has the highest national prevalence (44 %)^(^ ^18^ ^)^, followed by Afghanistan (41 %)^(^ ^16^ ^)^ and Nepal (41 %)^(^ ^20^ ^)^, India (39 %)^(^ ^21^ ^)^, Bangladesh (36 %)^(^ ^19^ ^,^ ^22^ ^)^ and Sri Lanka (13 %)^(^ ^23^ ^)^.

Among women of reproductive age in Afghanistan, 23 % suffer from Zn deficiency, measured as serum Zn concentration <60 µg/dl^(^ ^16^ ^)^. There is a higher prevalence among women in Bangladesh (57 % of non-pregnant, non-lactating women) and Pakistan (41 % of non-pregnant women). Variation in Zn deficiency by region and province among women is comparable to that seen among children. In Pakistan, there was no change in the prevalence of Zn deficiency among non-pregnant women between 2001 and 2011. This lack of progress is consistent with Wessells and Brown’s reported population Zn inadequacy based on estimates derived from the food supply^(^ ^6^ ^)^.

Comparisons across indicators of Zn deficiency should be done cautiously given that each is measuring a different component of Zn status^(^ ^24^ ^)^. For instance, serum Zn concentration is an indicator specific to biological Zn status, while stunting is a condition caused by several factors and not specific to Zn status, and estimated population Zn inadequate intake is based on Zn availability in the food supply for a population and thus not specific to individuals or sensitive to specific target groups within a population such as children or women^(^ ^25^ ^)^.

Among the four countries that did have data on the three or four indicators discussed, there is no clear pattern in indicators that may be over- or underestimating Zn deficiency and most indicators suggested that the high levels of Zn deficiency are a public health concern, although there were some inconsistencies. All four indicators suggest moderate to very high Zn deficiency in the population in Bangladesh and Pakistan. This includes estimated population Zn inadequate intake >15 %, low serum Zn among children and women each >20 %, and high or very high stunting at >30 %. Estimates were less consistent in Afghanistan and Sri Lanka. In Afghanistan, estimated population Zn inadequate intake and low serum Zn among women suggest a public health concern and stunting indicates a very high prevalence at 41 %, but conversely low serum Zn among children did not indicate a public health concern.

While stunting and low serum Zn among children in Sri Lanka were both low and do not indicate a current public health concern, the estimated population with inadequate Zn intake is 47 %, which is the highest in the region and indicative of a high concern. However, this may be due to methodological limitations of the indicator (estimated population inadequate Zn intake), where the researchers had to make assumptions regarding levels of Zn and phytate of food items according to which form they are most typically consumed in a given country. Wheat in particular was treated differently in Sri Lanka, where it is mostly consumed as white flour, and the rest of South Asia, where it is typically consumed as a whole grain. Dietary diversification and Zn supplementation and fortification are approaches to preventing Zn deficiency. In order for dietary-based approaches to succeed, the food supply must change. In an analysis of micronutrient intakes among children and women in rural Bangladesh, energy intake and dietary diversity explained 71–76 % of the variance in the mean probability of adequacy for the eleven micronutrients evaluated (including Zn), suggesting that increased diet diversification and energy intake could substantially reduce micronutrient deficiencies in this context^(^ ^26^ ^)^.

Efforts are underway to improve the availability of Zn in across South Asia via enrichment and fortification. In Bangladesh, approaches have been identified to improving Zn content in rice as part of the Nutritious Rice Value Chain innovation project. The most promising approach identified was to fortify rice during the soaking process^(^ ^27^ ^)^. Biofortification of rice has also been explored. One modelled estimate suggests that rice biofortification in Bangladesh could reduce the prevalence of Zn deficiency among children by 59 % (from 22 to 9 %)^(^ ^28^ ^)^.

### Iodine

While all South Asian countries have a median urinary iodine concentration (UIC) within an acceptable public health range at the national level, meaning that insufficient iodine status is not considered to be a public health problem at the country level, more than 20 % of each country’s population has a UIC < 100 µg/l, indicating mild or moderate insufficient iodine at an individual level^(^ ^4^ ^)^.

Typical indicators of iodine status at the population level include median UIC, prevalence of UIC < 100 µg/l, percentage of households using iodized salt and percentage of households with adequately iodized salt. Indicators of iodine status vary by region, such as use of iodized salt in Afghanistan, percentage of adequately iodized salt in India and Nepal, median UIC among women and school-aged children in Pakistan, and median UIC among school-aged children in Bangladesh. In Afghanistan, iodized salt is used by 74 % of the population, ranging from 32 % in Badakhshan to 98 % in Parwan and Khost^(^ ^16^ ^)^. In India, 78 % of households tested have adequately iodized salt based on the National Iodine and Salt Intake Survey 2014–15, which varied by region (62 % in the south *v*. 87 % in the north) and has increased from 51 % across India in 2005–06^(^ ^29^ ^,^ ^30^ ^)^. Approximately 73 % of households with a child <5 years in Nepal have adequately iodized salt (91 % in urban areas *v*. 71 % in rural regions)^(^ ^20^ ^)^.

Interestingly, median UIC among women and school-aged children in Pakistan is lower in urban (women, 96 µg/l; school-aged children, 119 µg/l) than in rural regions (women, 113 µg/l; school-aged children, 134 µg/l) and varies widely across provinces among women (from 63 to 149 µg/l) and school-aged children (from 62 to 160 µg/l)^(^ ^18^ ^)^. In Bangladesh, median UIC is higher among boys than girls (166·7 *v*. 122·7 µg/l), a difference that is wider in urban regions (167·6 *v*. 106·7 µg/l) and narrower in the slums (173·5 *v*. 172·3 µg/l)^(^ ^19^ ^)^. UIC among children increases with wealth index, but not among women^(^ ^19^ ^)^.

When we compare the pattern of median UIC and percentage of households consuming iodized salt in 2015 across the region, we find that while the coverage of iodized salt is lowest in Afghanistan relative to the rest of the region, median UIC is the third highest in the region after Nepal and Bhutan. Sri Lanka, on the other hand, has the highest coverage of iodized salt among households in the region, but is in the bottom three countries in the region for median UIC, along with the Maldives and Pakistan^(^ ^4^ ^)^.

**Fig. 2:**
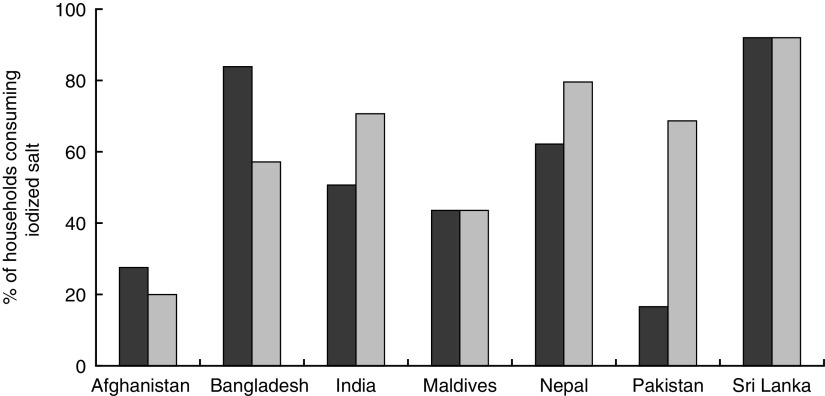
Percentage of households in South Asia consuming iodized salt in 2012 (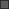) and 2015 (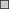), by country. (Data from Andersson *et al*.^(^ ^4^ ^)^ and the Iodine Global Network’s global iodine scorecard 2014–2015^(^ ^66^ ^)^)

Most South Asian countries, excluding Bhutan, Maldives and Pakistan, have established national policies for mandatory salt iodization (Table 1). When the Iodine Global Network’s iodine nutrition scorecards from 2012 and 2015 are compared, it is clear that progress towards increased iodized salt consumption has been made in India, Nepal and Pakistan, with a substantial increase in Pakistan (Fig. 2). Pakistan has been working towards an Iodine Deficiency Disorder Control Act, which has yet to pass. Given the progress Pakistan has already experienced in increasing iodized salt consumption, the potential for additional improvements from policy commitment is encouraging.

**Table 1:** Summary of salt iodization policies in South Asia. (Data from Bégin and Codling^(^ ^31^ ^)^)

Country	Year policy was initiated	Policy type	Policy objective	Household coverage (% using iodized salt)^(^ ^66^ ^)^
Afghanistan	2007	Under an existing law/act	Non-iodized salt is allowed	20·4
Bangladesh	1989	Stand-alone	Non-iodized salt banned or only iodized salt allowed	57·6
Bhutan	1984*	–	All salt must be iodized (interpreted)	91·0^(^ ^67^ ^)^
India	1998	Under the Food Act	Non-iodized salt banned or only iodized salt allowed	71·1
Maldives	–	–	All salt must be iodized (planned)	44·0
Nepal	1996	Stand-alone	All salt must be iodized	80·0
Pakistan	No national	Stand-alone†	Non-iodized salt banned or only iodized salt allowed (drafted)	69·1
Sri Lanka	1995	Under the Food Act	Non-iodized salt banned or only iodized salt allowed	92·4

* In 1984 a National Policy, Strategy and Plan of Action to Control Iodine Deficiency Disorder (IDD) was started and the IDD Control Program implemented^(^ ^67^ ^)^.

† IDD Control Bill of 2009 was drafted but not passed.

Bangladesh was the first country in the region to instate a policy (1989). Countries without a national policy mandating salt iodization have drafted some form of a bill or plan to address iodization. Afghanistan, Bhutan, Maldives, Nepal and Pakistan have or have planned legislation that requires iodization of salt for human and animal consumption and salt in processed foods. Bangladesh does not require iodization of processed foods and India does not require iodization of processed food or salt for animal consumption^(^ ^31^ ^)^. In Afghanistan, where non-iodized salt is allowed, the percentage of households consuming iodized salt is the lowest in the region (20 %), which may be a reflection of how recently the policy in Afghanistan was enacted (2007).

### Anaemia and iron status

**Fig. 3:**
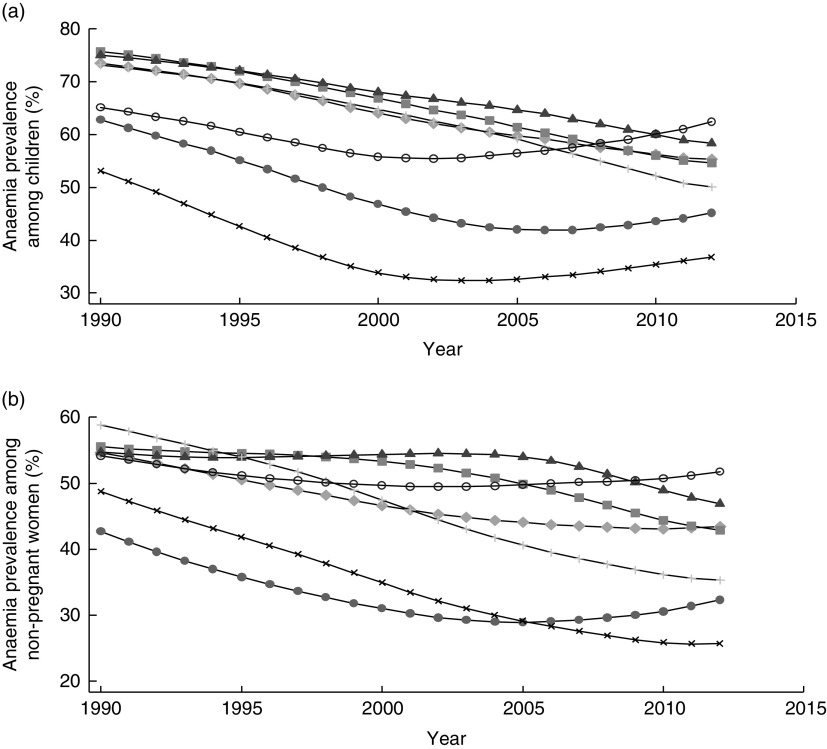
Prevalence of anaemia across time (1990–2012) among (a) children 6–59 months old and (b) women of reproductive age in South Asia, by country: 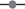, Afghanistan; 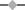, Bangladesh; 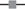, Bhutan; 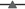, India; 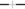, Nepal; 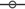, Pakistan; 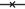, Sri Lanka. (Data from Stevens *et al*.^(^ ^3^ ^)^)

Fe deficiency is the leading cause of anaemia among men and women in South Asia, accounting for an estimated half the cases of anaemia^(^ ^32^ ^)^. Modelled data suggest that little progress has been made in reducing anaemia among children 6–59 months old and non-pregnant women in many South Asian countries since 1990 (Fig. 3)^(^ ^3^ ^)^. Similarly, the burden of Fe-deficiency anaemia has changed little over the years^(^ ^33^ ^)^. Slow progress towards reducing anaemia has been made in Bangladesh, Bhutan, India and Nepal. However, all South Asian countries, with the exception of Sri Lanka, have a prevalence of anaemia among children 6–59 months old that indicates a severe public health problem (≥40 %); in Sri Lanka, the problem is classified as ‘moderate’ (20·0–39·9 %)^(^ ^34^ ^)^. Similarly, not one of South Asia’s nations is ‘on course’ to meet the 2025 target adopted by the World Health Assembly of reducing anaemia among women of reproductive age by 50 %^(^ ^14^ ^)^.

A review of nationally representative data on anaemia in South Asia published in 2012 brought to light new national nutrition surveys from Afghanistan^(^ ^16^ ^)^, Bangladesh^(^ ^19^ ^)^, Bhutan^(^ ^35^ ^)^ and Sri Lanka^(^ ^23^ ^)^. In Afghanistan, the prevalence of anaemia among women increased from 38 % in 2004 to 40 % in 2011, and among children from 24 to 45 %^(^ ^16^ ^)^; similarly, in Pakistan, the prevalence of anaemia among non-pregnant women increased from 28 % in 2001 to 50 % in 2011, and among children 0–59 months it increased from 51 to 62 %^(^ ^18^ ^,^ ^36^ ^)^. By contrast, the prevalence of anaemia among non-pregnant women and children <5 years in Bhutan decreased between 2003 and 2015 from 55 to 36 % and from 81 to 44 %, respectively^(^ ^35^ ^)^. In Sri Lanka, the prevalence of anaemia in 2012 among children <5 years remained low at 15 %^(^ ^23^ ^)^. There is a lack of data on anaemia among adolescent girls, another important at-risk group. However, the data available indicate that 69 % of adolescent girls are anaemic in India and 31 % in both Afghanistan and Bhutan^(^ ^16^ ^,^ ^21^ ^,^ ^35^ ^)^.

The Bangladesh Demographic and Health Survey 2011 reports that the prevalence of anaemia among children <5 years and women of reproductive age is 51 and 42 %, respectively^(^ ^37^ ^)^, which is greater than the prevalence of anaemia among children <5 years (31 %) and non-pregnant non-lactating women (26 %) reported in the 2011–2012 National Micronutrients Status Survey^(^ ^19^ ^)^.

In India, Bangladesh and Nepal, anaemia is more prevalent among rural than among urban populations, whereas in Pakistan there is little difference by rural/urban residence (see online supplementary material, Supplemental Fig. 1). Large sub-national differences are observed within Bangladesh, India and Nepal. States in central India have a prevalence of anaemia among children >70 %, while Bihar, in the north-east, has the highest prevalence of anaemia at 78 %^(^ ^21^ ^)^. In Bangladesh, the Rangpur and Barisal divisions have the highest prevalence of anaemia among children and women and in Nepal, the highest prevalence of anaemia among children is found in the *terai* (50 %), although levels in the mountains (48 %) and hills (41 %) are also high^(^ ^20^ ^)^.

Despite the high prevalence of anaemia in South Asia, and the approximation that half of anaemia in South Asia is due to Fe deficiency^(^ ^32^ ^)^, nationally representative data on women and children <5 years from Afghanistan (2013), Bangladesh (2011–2012) and Sri Lanka report a prevalence of Fe deficiency between 24 and 26 % in Afghanistan, 7 and 11 % in Bangladesh and of 34 % in Sri Lanka, and a prevalence of Fe-deficiency anaemia of 14 % in Afghanistan, between 5 and 7 % in Bangladesh and 7 % in Sri Lanka^(^ ^16^ ^,^ ^17^ ^,^ ^19^ ^)^. Roughly 27 % of non-pregnant women and 44 % of children in Pakistan have low ferritin concentration^(^ ^18^ ^)^. In a recent survey in rural Bangladesh, a high prevalence of anaemia was reported (57 %) where Fe deficiency was absent^(^ ^38^ ^)^, suggesting other causes of anaemia. In the 2012 Sri Lankan National Nutrition and Micronutrient Survey, 52 % of anaemic children were Fe deficient, 13 % had haemoglobinopathies and 4 % had acute infection^(^ ^17^ ^)^. In all contexts, it is crucial to understand the cause of anaemia to know how to prevent and treat it. In response to a 75 % prevalence of anaemia among pregnant women in 1998, Nepal’s Government began the Intensification of the (national) Maternal and Neonatal Micronutrient Program in 2004 with support by UNICEF and the Micronutrient Initiative^(^ ^39^ ^)^. The programme targeted antenatal care visits, Fe and folic acid supplementation and deworming prophlyaxis during pregnancy and utilized the existing infrastructure of vitamin A supplementation (VAS) through female community health volunteers. Programme indicators suggested successful implementation and utilization and the prevalence of anaemia among pregnant women dropped to 42 % in 2006.

India’s efforts to address anaemia among adolescents are also notable. The Adolescent Girls’ Anaemia Control Programme provides Fe/folic acid tablets (weekly), nutrition counselling (monthly) and deworming prophylaxis (biannually) to adolescent girls through schools and *anganwadi* centres (village centres of India’s Integrated Child Development Services or ICDS programme). During the initial year of this programme, the prevalence of anaemia among adolescent girls declined by 24 %. The programme being since scaled up nationally under the name Weekly Iron and Folic Acid Supplementation Program (WIFS), covering both adolescent girls and boys^(^ ^40^ ^,^ ^41^ ^)^.

Despite efforts made to address anaemia and Fe deficiency, anaemia remains a severe public health problem in South Asia and requires additional large-scale efforts to address it. Targeted efforts to high-risk groups of this population and a focus on the underlying causes of anaemia in the different environments will be most effective.

### Vitamin A

**Fig. 4:**
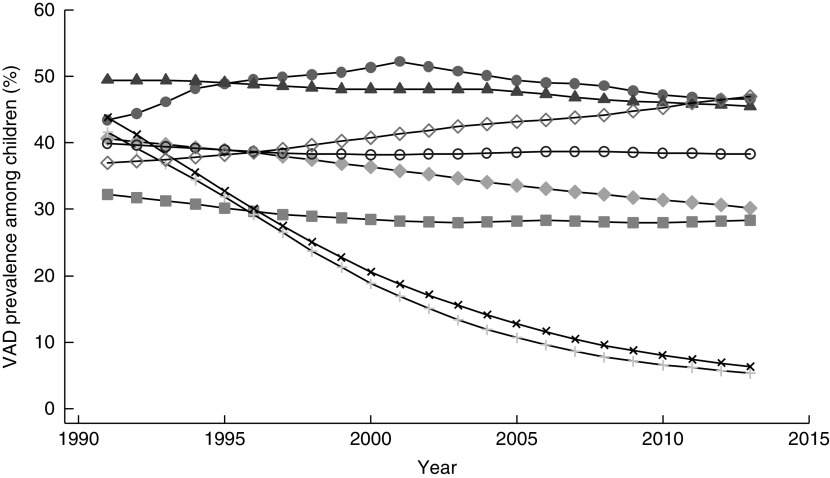
Prevalence of vitamin A deficiency (VAD) across time (1991–2013) among children 6–59 months old in South Asia, by country: 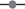, Afghanistan; 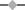, Bangladesh; 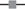, Bhutan; 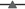, India; 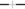, Maldives; 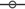, Nepal; 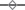, Pakistan; 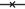, Sri Lanka. (Data from Stevens *et al*.^(^ ^2^ ^)^)

South Asia remains the world’s region with the greatest number of children affected by VAD. As with other deficiencies, little progress has been made towards reducing VAD between 1991 and 2013^(^ ^2^ ^)^. Disaggregated by country, modelled data indicate that Sri Lanka and the Maldives have achieved substantial reductions in VAD among children since 1991 and Bangladesh has made some progress (Fig. 4). In contrast, the prevalence of VAD among children in Afghanistan and Pakistan has increased, while Bhutan, India and Nepal have seen little improvement.

Both Sri Lanka and the Maldives have nationwide VAS programmes integrated with other services, as do other countries in the region. The Maldives experienced an increase in activity by the Department of Public Health and a greater focus on public health between the late 1990s and 2000s. The country’s government also maintains multisectoral coordination between the Departments of Education and Public Health and the country has experienced growth in development and infrastructure over the last decade, including health centres or hospitals on each island and progress in the transport sector with high-speed speedboats and boats, both of which could contribute to increased access to health care and affordable and nutritious foods. All these factors could have contributing roles to the Maldives’ success in reducing VAD. Thus, contextual factors that may alter access to health care and food should also be considered alongside VAS programmes with high coverage as countries aim to reduce VAD and other forms of malnutrition.

Based on the national nutrition survey 11 % of women of reproductive age are vitamin A deficient in Afghanistan^(^ ^16^ ^)^, 40 % of non-pregnant, non-lactating women in Bangladesh have VAD^(^ ^19^ ^)^, and 42 % of non-pregnant women and 46 % of pregnant women in Pakistan have VAD^(^ ^18^ ^)^. The most recent nationally representative data of VAD in Nepal are from 1998, at which time 17 % of women had low serum retinol^(^ ^42^ ^)^.

The coverage of VAS in South Asia increased from 58 % in 2003 to 71 % of children <5 years in 2005^(^ ^43^ ^,^ ^44^ ^)^. This varies by country from 45 % in Bhutan and 53 % in India to over 95 % in Afghanistan, Bangladesh, Nepal and Pakistan (Table 2). While coverage is an important performance indicator for VAS programmes, it does not necessarily reflect serum retinol and VAD levels of the population^(^ ^45^ ^)^; thus, as indicated by the example of the Maldives, additional commitments and progress aid in reducing VAD. Coverage data may also be an overestimation of actual coverage in contexts where countries’ self-report from administrative records such as tally sheets are being used^(^ ^46^ ^,^ ^47^ ^)^.

**Table 2:** Vitamin A supplementation coverage rate* in South Asia, by country, in 2013†. (Data from the World Bank^(^ ^68^ ^)^)

Country	Coverage (%)
Afghanistan	97
Bangladesh	97
Bhutan	45
India	53
Maldives	76
Nepal	99
Pakistan	99
Sri Lanka	89

* Percentage of children 6–59 months old who receive at least two doses of vitamin A in the previous year.

† Data for Pakistan are from 2012.

One strategy that successfully increased VAS coverage is the pairing of this programme with existing National Immunization Days. In the late 1990s, the WHO recommended that VAS be delivered as part of the National Immunization Days, which was implemented successfully in many countries^(^ ^44^ ^)^. Horton *et al*. point out the success of Bangladesh in achieving high VAS coverage and reaching the hard-to-reach populations through combining routine health services to target 6- to 11-month-olds and Child Health Days (also called National Vitamin A Plus Campaign) to target 12- to 59-month-olds, an approach also used in India^(^ ^48^ ^)^.

Supplementation of pregnant or lactating women with vitamin A is not standard across South Asia, although it has been implemented in Nepal through community health volunteers^(^ ^48^ ^)^. This can be another approach to reaching infants *in utero* or soon after birth and has the potential to affect vitamin A stores and survival, although the primary purpose of supplementing pregnant women has been to improve maternal and birth outcomes. Fortification is also an approach to addressing VAD. In Afghanistan, vegetable oil and ghee have been fortified with vitamins A and D at the national level, a programme that started in 2012, while in Rajasthan state in India oil and milk have been fortified since 2007 with Fe, folic acid, vitamins A, B_12_ and D, and Bangladesh has required vegetable oil fortification with vitamin A since 2013^(^ ^49^ ^–^ ^51^ ^)^. Dietary diversification is yet another approach to increasing vitamin A intake and has been shown to have a significant positive association with serum retinol concentrations^(^ ^52^ ^)^. As an intervention, this approach first requires that diverse diets be available, accessible and affordable.

## Policy implications of hidden hunger

South Asia’s policy makers must urgently address a multifaceted challenge when it comes to promoting improved nutrition. They need to make appropriate large-scale investments of various kinds that can cost-effectively tackle widespread undernutrition, try to prevent – or at least contain – the spread of child, adolescent and adult overweight and obesity, and also pay much more attention to resolving the very serious micronutrient deficiencies that affect millions of people. Evidence-based nutrition interventions exist that can be implemented, scaled up or modified, such as those highlighted in the 2008 and 2013 *Lancet* series on Maternal and Child Nutrition (Box 1)^(^ ^53^ ^,^ ^54^ ^)^. Identifying which interventions are effective in different settings in South Asia is crucial and, as noted by Bhutta *et al*., specifying appropriate delivery platforms is just as important as the content of the package delivered^(^ ^54^ ^)^. For example, the female community health volunteers in Nepal have been a successful platform to deliver behaviour change nutrition counselling and vitamin A and Fe supplements, while the Adolescent Girls’ Anaemia Control Programme in India has been able to achieve large coverage through using schools and *anganwadi* centres to reach both adolescent girls in school and out of school, at a cost of $US 0·40 per adolescent^(^ ^55^ ^)^.

Box 1 Nutrition interventions to target maternal and child nutrition. (List from 2008 and 2013 *Lancet* series on Maternal and Child Nutrition^(^ ^53^ ^,^ ^54^ ^)^)

Maternal and birth outcomes

● Fe/folate supplementation

● Maternal supplements of multiple micronutrients

● Maternal iodine through iodization of salt

● Maternal Ca supplementation

● Interventions to reduce tobacco consumption or indoor pollution

● Maternal supplementation with balanced energy and protein

Newborn babies

● Promotion of breast-feeding

● Delayed cord clamping (especially for preterm infants)

● Vitamin A supplementation

● Vitamin K administration

Infants and children

● Promotion of breast-feeding

● Behaviour change communication for improved complementary feeding

● Fe supplementation of children

● Zn supplementation of children

● Vitamin A supplementation or fortification

● Multiple micronutrient supplementation of children including Fe

● Universal salt iodization

Disease prevention and management

● Handwashing or hygiene interventions

● Treatment of severe acute malnutrition

● Zn in management of diarrhoea

● Deworming in children

● Malaria prophylaxis in children

● Intermittent preventative treatment of malaria in pregnancy

Supplementation with single or multiple micronutrients accounts for many well-established nutrition-specific interventions. VAS of children <5 years, for example, has successfully reduced all-cause mortality, diarrhoea-related mortality, and diarrhoea and measles incidence^(^ ^54^ ^)^. Nepal’s National Vitamin A Program is recognized by the global community as a success. Established in 1993, and reaching almost 88 % coverage nationally in 2006, this programme revitalized the Female Community Health Volunteer programme in Nepal, which has since been used as a platform for other nutrition interventions^(^ ^56^ ^)^. The cost to provide two vitamin A capsules to a child each year was $US 1·25 in 2000, suggesting high cost-effectiveness^(^ ^57^ ^)^. Large-scale VAS programmes tend to miss ‘hard-to-reach’, vulnerable subgroups^(^ ^48^ ^)^. In a 2014 analysis of India’s VAS programme coverage data, the percentage of poor children missed by the programme decreased, although children from scheduled castes and scheduled tribes were still missed^(^ ^58^ ^)^. Targeting these groups will be a key priority for reaching greater coverage, equity and population-level impact.

Implementation research into how existing programmes might be modified to fill current gaps is needed. In the case of India’s Universal Salt Iodization Program, such a review has been completed and five areas of challenge were identified: (i) ensuring political commitment; (ii) forming partnerships and coalitions; (iii) ensuring availability of adequately iodized salt; (iv) strengthening the monitoring system; and (v) maintaining continuous advocacy, education and communication^(^ ^59^ ^)^.

Efforts aimed at enrichment and fortification with Fe, vitamin A, Zn and other micronutrients in staple South Asian foods are also underway, with the potential to substantially reduce micronutrient deficiencies in this region^(^ ^49^ ^,^ ^50^ ^)^; biofortification approaches are also being explored^(^ ^28^ ^)^. Review of the wheat and edible oil fortification programmes in Afghanistan and Pakistan recommends supporting efforts to make wheat flour fortification mandatory, building standardized processes and regulatory framework within and across countries, clearly defining roles and responsibilities for monitoring and enforcement, reducing the tax on premix, and incentivizing the industries to comply with fortification^(^ ^60^ ^)^. As well, the Food Safety and Standards Authority of India has made strides in developing the Food Safety and Standards (Fortification of Foods) Regulations, 2016 to mandate fortification of specific micronutrients to key foods.

Interventions that promote breast-feeding, timely and age-appropriate complementary feeding and dietary diversity are increasingly important interventions that have the potential to combat not only micronutrient deficiencies and undernutrition along with stunting and wasting, but also the growing concern of overweight and obesity. For example, breast-feeding not only provides the most cost-effective nutritionally complete food to infants younger than 6 months and continues to be a key source of nutrients during the first 2 years of life, there is also evidence that breast-feeding could be protective against obesity^(^ ^61^ ^,^ ^62^ ^)^. Furthermore, the long-term cognitive and economic impacts of breast-feeding are also documented^(^ ^63^ ^)^. Similarly, promotion of timely and appropriate complementary foods for children and increasing diet diversity for children aged 6–23 months, women and the general population also have the potential to be effective and sustainable solutions to hidden hunger^(^ ^26^ ^,^ ^54^ ^)^. There are recent examples of how infant and young child feeding practices can be improved at scale^(^ ^64^ ^)^. Important challenges to these food-based interventions include the accessibility (physical and financial), at the country, household and individual level, and acceptability.

Greater investments in scaling up evidence-based nutrition interventions and exploring promising approaches are crucial. Analysis modelling the impact of scaling up the coverage of ten nutrition-specific interventions to 90 % in thirty-four countries bearing a high burden of child malnutrition shows such action would reduce child mortality globally by 15 % and the cost would be approximately $US 9·6 billion per year^(^ ^54^ ^)^. These interventions included: salt iodization for the general population; multiple micronutrient supplementation in pregnancy; Ca supplementation in pregnancy; energy–protein supplementation in pregnancy; VAS in childhood; Zn supplementation in childhood; breast-feeding promotion, complementary feeding education; complementary food supplementation; and management of severe acute malnutrition. The cost of scaling up the five micronutrient-specific supplementation interventions would cost $US 3·7 billion of that total.

While these investments may seem substantial, the cost of not investing is far greater. Stein and Qaim calculated the human and economic cost of hidden hunger for India alone, where an estimated 9·3 million disability life-years are lost due to Fe-deficiency anaemia and Zn, vitamin A and iodine deficiencies, accounting for 0·8 to 2·5 % of India’s Gross Domestic Product^(^ ^12^ ^)^. Based on India’s Gross Domestic Product in 2014, this translates to $US 16·5 to 51·7 million. While there is a lack of data to make similarly detailed economic estimates for all countries, the economic benefit of addressing micronutrient deficiencies in South Asia is clear.

In addition to targeted nutrition-specific interventions, policy makers need to bring actions from other sectors to bear on the problems of hidden hunger. That is, there is ‘enormous untapped potential’ in South Asia for governments to invest in nutrition-sensitive interventions in the related sectors of agriculture, education, water and sanitation, social protection and infrastructure development which can each help address different underlying determinants of malnutrition^(^ ^65^ ^)^. Different governments must make locally appropriate choices about which sectors and expenditure levels will be needed to achieve desirable results in line with local and global nutrition targets.

## Conclusions

The most recent data available confirm that progress towards controlling iodine deficiency disorders is promising, with adequate iodine status in most countries. On the other hand, reductions in Zn deficiency, anaemia and VAD in South Asia remain slow, and deficiencies are at levels that require immediate policy attention. While the link between Fe deficiency and anaemia should not be disregarded, more attention is needed to accurately define the aetiology of anaemia in South Asia, such as what has been done in Sri Lanka and what is underway in Bangladesh and Nepal, and cause-specific interventions should be implemented. Data on Fe deficiency specifically are necessary, but significant gaps remain in our understanding of the location, prevalence, impact and causes of all micronutrient deficiencies in South Asia. Given the need for a large increase in public investments across the region to address the scale and complexity of these problems, high-quality disaggregated data on status and trends are needed, as is empirically based evidence of successful policies and programmes that can achieve cost-effective change at scale. Many more targeted interventions of information, services and resources are needed to meet the needs of the hard-to-reach and most high-risk populations, but these must be combined with nutrition-sensitive actions and food system approaches to sustainably secure the nutrient needs of South Asia’s growing population.

## Supplementary material

For supplementary material accompanying this paper visit https://doi.org/10.1017/S1368980017003202. click here to view supplementary material
