# Supplementary material for: Hidden hunger in South Asia: a review of recent trends and persistent challenges
Source: Public Health Nutr. 2017 Dec 19;21(4):785–95. doi: 10.1017/S1368980017003202 (PMC5851053; doi:10.1017/S1368980017003202)
Supplement: Supplementary file 1 [file S1368980017003202sup001.docx]

**Hidden hunger in South Asia: Supplementary Material**

**Supplemental Fig. 1.** Prevalence of anemia in South Asia, by urban and rural [Stratified data for Afghanistan was not available]

<5, children 0 to 59 months old; WRA, women of reproductive age
